# IgA defects in CVID lead to bacterial translocation, increased serum γ-interferon, and BAFF

**DOI:** 10.70962/jhi.20250080

**Published:** 2026-01-13

**Authors:** Hsi-en Ho, Lin Radigan, Eric Meffre, Charlotte Cunningham-Rundles

**Affiliations:** 1 Department of Medicine, Icahn School of Medicine at Mount Sinai, New York, NY, USA; 2 Division of Immunology and Rheumatology, Department of Medicine, Stanford University School of Medicine, Stanford, CA, USA

## Abstract

Common variable immunodeficiency (CVID) is a primary antibody defect that leads to frequent infections, but inflammatory complications appear in 30–50%, leading to increased morbidity and mortality. We have previously shown that circulating bacterial 16S ribosomal DNA (rDNA), originating from gut commensals, is significantly increased in the serum of patients with CVID with inflammatory conditions (P = 0.0007). Here we examined the relationships between serum 16S ribosomal DNA (rDNA), isotype-switched memory (SM) B cells, serum IgA, IgA+ SM B cells, serum IFN-γ, and serum B cell–activating factor (BAFF). We found a significant inverse correlation between serum 16S ribosomal DNA (rDNA) concentrations and the numbers of isotype SM B cells, IgA+ SM B cells, and serum IgA levels in our large cohort, suggesting that loss of IgA in the mucosal barrier permits bacterial transcytosis. Loss of SM B cells and lower serum IgA concentrations were both associated with increased serum IFN-γ, as well as increased CXCL9 and serum BAFF concentrations. Serum BAFF was also significantly associated with IFN-γ levels and inversely correlated with baseline serum IgA. We conclude that loss of IgA, accompanied by mucosal defects in CVID, may permit bacterial transcytosis, resulting in excessive IFN-γ and BAFF production, both of which promote autoimmune and inflammatory complications in this immune defect.

## Teaser

Inflammatory complications occur in many subjects with common variable immunodeficiency. We show that microbial bacterial 16S ribosomal DNA, significantly increased in the serum of these subjects, is associated with loss of IgA, leading to excess IFN-γ and increased serum B cell–activating factor (BAFF), both of which promote autoimmunity and inflammation.

## Introduction

Common variable immunodeficiency (CVID), the most prevalent symptomatic primary immunodeficiency, is characterized by low levels of serum immunoglobulin (Ig) G, A, and/or M and a lack of specific antibodies after vaccination or disease exposure (1, 2, 3). Due to loss of protective antibodies, subjects with CVID are susceptible to recurrent, severe infections, commonly of the respiratory tract. However, noted for years is that up to 30–50% of CVID subjects may develop a variety of additional inflammatory or autoimmune conditions (4, 5), commonly termed “noninfectious complications.” These conditions include autoimmunity (especially thrombocytopenia and hemolytic anemia), interstitial lung disease, lymphoid hyperplasia, enteropathy, nodular regenerative hyperplasia of the liver, systemic granulomatous disease, and in some, lymphoid malignancy (4, 5, 6, 7). These complications pose difficulties for treatment, as standard immune globulin therapy provides neither a treatment nor a prevention of these conditions, and immune suppression in immune deficiency is generally to be avoided. Taken together, these inflammatory conditions lead to an 11-fold increased morbidity and mortality in CVID (5, 8).

One of the most useful methods of subdividing CVID subjects is by considering the number of isotype-switched memory (SM) B cells in circulation. Subjects with the lowest numbers of these cells have been shown to be patients with the greatest risk of inflammatory complications (9, 10), but the reasons for this have not been clear. While recent investigations have demonstrated that 25–30% of subjects have one of a number of genetic defects, this work has not supplied a better understanding nor suggested a therapeutic option for the majority of subjects (11, 12, 13).

On investigating inflammatory pathways that might distinguish CVID subjects, we previously demonstrated a marked upregulation of IFN-γ pathways by mRNA transcriptional profiling (14, 15). The IFN signature was also associated with an expanded population of IFN-γ–, IL-17A–, and IL-22–positive innate lymphoid cells (ILCs) in peripheral blood and also in gastrointestinal mucosal and lung tissues of CVID subjects (15). As ILCs play an important role in host-commensal homeostasis (16), we suggested that their excessive activity and/or proliferation could contribute to systemic, mucosal, and organ-specific inflammation in CVID. However, the stimulus underlying these immune responses has remained unknown. We have recently shown that circulating bacterial 16S ribosomal DNA (rDNA), primarily from gut commensals, is significantly increased in CVID serum (P < 0.0001), but especially in those subjects with low numbers of SM B cells and increased numbers of inflammatory conditions (P = 0.0007) (17). These bacterial ribosomal DNA (rDNA) levels were also significantly associated with increased serum IFN-γ, TNF-α, IL-6, soluble CD14, lipopolysaccharide-binding protein, and the mucosal markers of gastrointestinal mucosal leakage, zonulin and intestinal fatty acid binding protein (intestinal fatty acid binding protein (I-FABP)) (17, 18).

In mice and humans, secretory IgA and IgM have been shown to limit bacterial translocation from mucosal compartments (19, 20, 21, 22, 23). Here we show that the levels of bacterial ribosomal DNA (rDNA) in CVID serum are inversely correlated with the numbers of isotype SM B cells, the numbers of circulating IgA^+^ SM B cells, and residual serum IgA concentrations. We find that the levels of bacterial DNA are closely associated with loss of serum IgA and also with the levels of serum IFN-γ and B cell–activating factor (BAFF). In previous work, we showed that the serum of subjects with CVID contained high BAFF (24, 25), and separately, that increased serum BAFF was correlated with increasing B cell–rich lymphocytic infiltrates in the lung in CVID (26). Based on the current observations, we suggest that the loss of IgA, along with mucosal defects, permits gastrointestinal bacterial transcytosis, which then leads to the production of both of these inflammatory signals.

## Results

### Phenotypes and genetics

55 of the 114 subjects studied here (23 females and 32 males) (48%) had experienced one or more “noninfectious” complications; 31 had had one or more episodes of  immune thrombocytopenia (ITP), in some cases also hemolytic anemia, at the same or different intervals; 15 of these had interstitial lung disease, 16 had biopsy-identified granulomatous disease, 10 had marked lymphoid hyperplasia, 14 have significant enteropathy, 6 had severe liver disease, 3 had lymphoma, and 7 have had a splenectomy. Of the 114 subjects, 32 had one or more genetic mutations previously associated with CVID or considered to be pathogenic (28% of the group) (Table 1). In most cases, these were heterozygous, but one was homozygous. One subject had two pathogenic heterozygous mutations associated with CVID. Of the 32 subjects with known gene mutations, 19 had experienced one or more of the inflammatory complications; however, 14 other subjects (with no identified gene mutations) also had one or more of these complications.

**Table 1.** Gene mutations in patients

1	CYBA c.70G>A (p.Gly24Arg)
2	IKZF1. p.Arg162Trp
3	INO80. p.Arg1281Gln
4	INO80. p.Leu205Arg
5	INO80. p.Val1108Gly
6	IRF2BP2. p.Pro118Ser
7	IRF2BP2. p.Pro127Ser
8	LPIN2. p.Asn833*
9	NFKB1. essential splicing
10	NFKB1. essential splicing
11	NFKB1. p.Leu615Phe
12	NFKB1. p.Gln199*
13	NFKB1. p.Phe459fs
14	NFKB2. p.His98Asn; TNFRSF13B p.Cys104Arg
15	PI3KCD Glu1021Lys
16	PIK3R1. start_gained
17	PMM2. p.Arg141His
18	SPI1. p.Gly156Arg
19	STAT3. p.Arg246Gln
20	STAT3. p.V353F
21	STXBP2. p.Gly541Ser
22	STXBP2. p.Pro345Leu
23	TACI p. Cys172Phe
24	TACI. p.Leu171Arg
25	TNFRSF13B p.Leu69fs
26	TNFRSF13B p.Ile87Asn
27	TNFRSF13B p.Cys104Arg
28	TNFRSF13B p.Cys104Arg homozygous
29	TNFRSF13B p.Ala181Glu
30	TNFRSF13B p.Arg202Cys
31	TNFRSF13B p.Cys104Arg
32	TNFRSF13B p.Lys188del

### Bacterial DNA and isotype SM B cells

**Figure 1.**
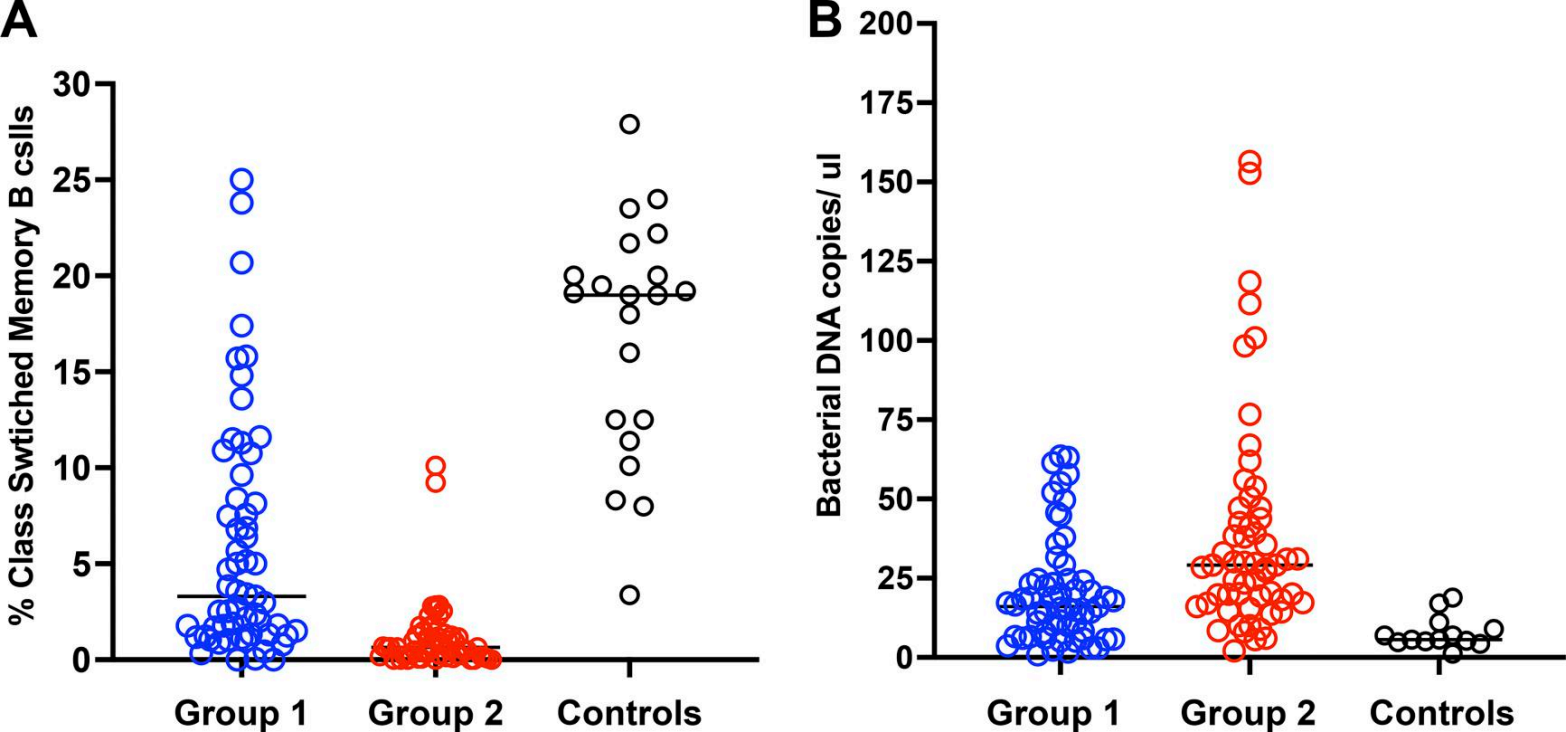
**Very low numbers of isotype switched memory B cells (SMB) in patients with CVID, is associated with the development of inflammatory complications. (A)** Isotype-switched member B cells and bacterial DNA in patients and controls: The percentage of SM B cells of 57 CVID subjects with 2% or fewer was compared to 59 CVID subjects with more than this number, or 21 normal controls, showing highly significant differences between these groups (Mann–Whitney test P = 0.001). **(B)** Increased bacterial DNA in subjects with inflammatory complications: Examining 59 CVID subjects with inflammatory conditions as compared to the 57 other CVID subjects without these conditions (or controls), showing highly significant differences between these groups (Mann–Whitney test P = 0.0008).

**Figure 2.**
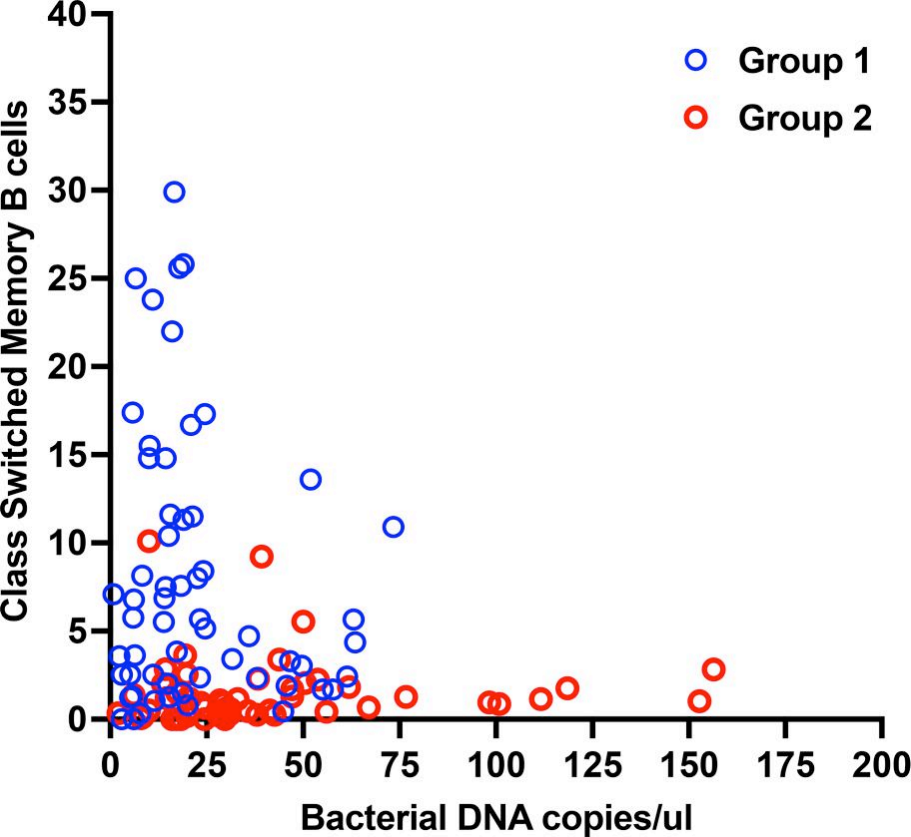
**Isotype-switched member B cells are inversely related to bacterial DNA in patients.** The numbers of isotype SM B cells for the 114 CVID patient cohort were significantly and inversely related to the amount of bacterial DNA for 90 CVID samples (Spearman r, two tailed test, P = 0.02; r = −0.31). Subjects in Group 1 (no inflammatory complications) are distinguished from those with these complications (Group 2).

A useful method of subdividing CVID subjects has been by considering the number of SM B cells in circulation, as subjects with the lowest numbers of these cells are known to be patients with the greatest risk of inflammatory complications (9, 10). This is illustrated for this cohort, examining the 55 CVID subjects with inflammatory complications (Group 2) as compared to 59 other CVID subjects without these medical issues (Group 1) or 21 normal controls, showing highly significant differences between the patient groups (P = 0.0001.) Fig. 1 A. We previously showed that the subjects with inflammatory complications also have substantially more bacterial ribosomal DNA (rDNA) in their serum than subjects with higher numbers of these SM B cells or normal controls (17). This is again illustrated for this cohort, examining here 55 CVID subjects with inflammatory conditions as compared to the 59 CVID subjects without these conditions (or controls), also showing highly significant differences between these same groups (P = 0.0008) (Fig. 1 B). The numbers of isotype SM B cells are also significantly and inversely related to the amount of bacterial DNA in the serum of the same patients, as shown for the total group (P = 0.038) (Fig. 2).

### Serum IgA, SM, and IgA+ SM B cells

**Figure 3.**
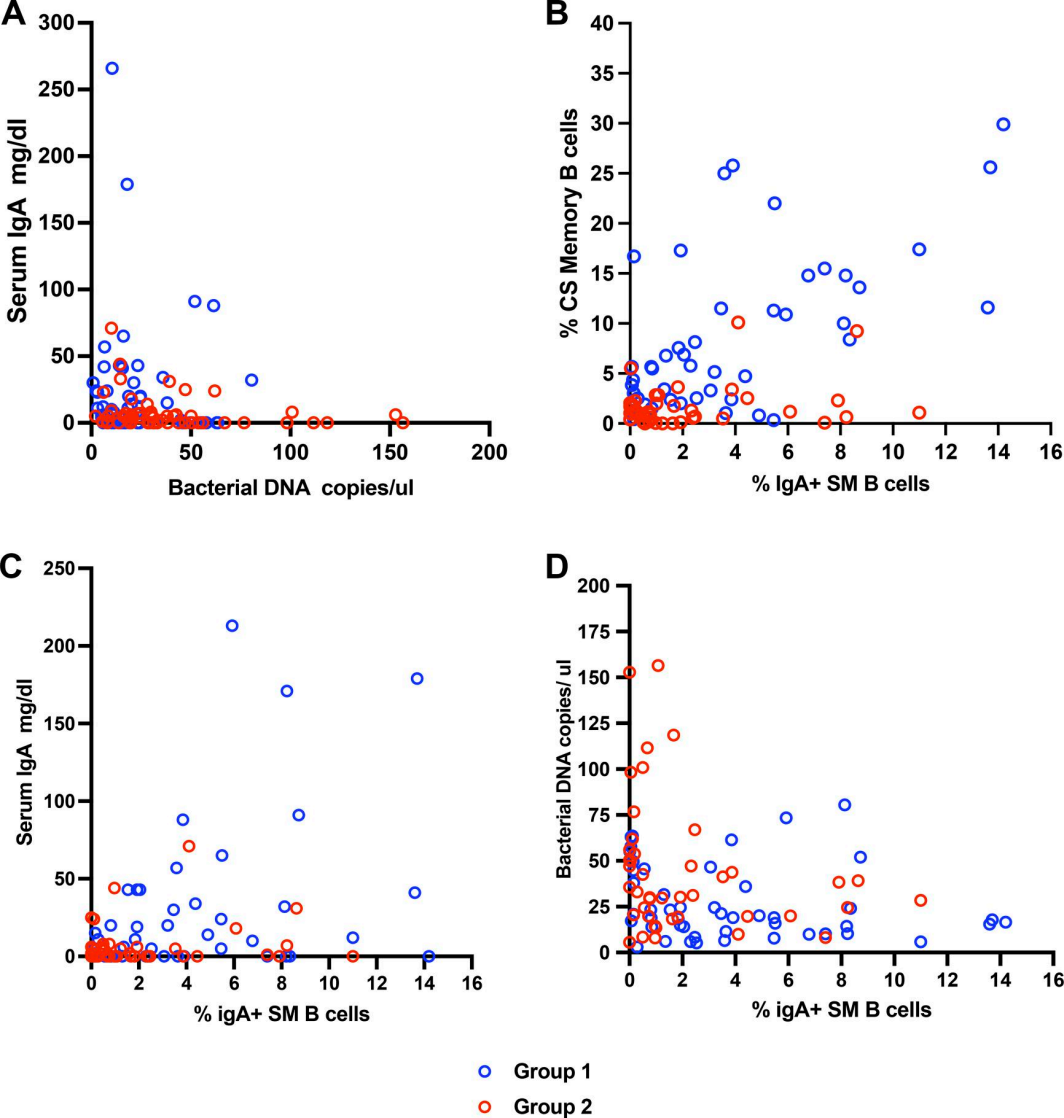
**A higher serum IgA is related to lower levels of bacterial DNA in blood. These subjects have more IgA+ switched B cells, and fewer inflammatory complications.** **(A)** Serum IgA and bacterial 16 ribosomal DNA (rDNA). Serum IgA is inversely related to the content of bacterial 16S ribosomal DNA (rDNA) in 111 paired samples (Spearman r, two tailed test P = 0.006; r = −0.3224). Subjects in Group 1 (no inflammatory complications) are distinguished from those with these complications (Group 2). **(B)** IgA+ SM B cells. The numbers of IgA+ SM B cells for 89 subjects were closely correlated with the numbers of total isotype SM B cells (Spearman r, two tailed test P = 0.002; r = −0.3265). Subjects in Group 1 (no inflammatory complications) are distinguished from those with these complications (Group 2). **(C)** IgA+ SM B cells and serum IgA. The numbers of IgA^+^ SM B cells are related to the baseline level of serum IgA of the same 92 patients (Spearman r, two tailed test P = 0.002; r = −0.322). Subjects in Group 1 (no inflammatory complications) are distinguished from those with these complications (Group 2). **(D)** IgA+ SM B cells and 16S ribosomal DNA (rDNA). The number of IgA^+^ SM B cells was also inversely related to the bacterial ribosomal DNA (rDNA) levels in the same 93 serum (Spearman r, P = 0.007; r = −0.2808). Again, CVID subjects in groups 1 and 2 are indicated.

As increased numbers of isotype SM B cell phenotype suggest that the B cells of these subjects may retain some capacity for normal function and may produce some serum IgA, we then tested this association. First, serum IgA concentrations for the entire group were overall correlated with the numbers of SM B cells in the same CVID subjects (P = 0.01). Second, suggesting a protective role of IgA in bacterial translocation, there was an overall inverse correlation between baseline serum IgA concentrations and the level of bacterial ribosomal DNA (rDNA) in the blood of 111 patients (P = 0.006). In Fig. 3 A, dividing CVID subjects into the two groups shows that Group 1 subjects (“infections only” phenotype, without inflammatory complications) had higher serum IgA levels and less bacterial DNA than Group 2 CVID subjects with inflammatory conditions. In contrast, there were no correlations between the serum concentrations of either serum IgG or baseline IgM and the amounts of circulating bacterial DNA of these same samples (P = 0.36; P = 0.33). We then examined if the numbers of circulating IgA+ SM B cells in CVID subjects might provide another useful biomarker for transcytosis of gut commensal bacteria. First, as one would expect, the numbers of IgA+ SM B cells were closely correlated with the numbers of total isotype class SM B cells in 94 CVID subjects, again shown in these groups (P = 0.0001) (Fig. 3 B), as well as with the baseline level of serum IgA concentrations of the same patients, (P = 0.002) (Fig. 3 C). The overall numbers of IgA^+^ SM B cells in blood were also inversely correlated with bacterial 16S ribosomal DNA (rDNA) levels in the same patient’s serum (P = 0.02). Fig. 3 D shows these data and also indicates the differences between these two cohorts, (Group 1) without as opposed to those with inflammatory complications (Group 2). We noted also that the CVID subjects with lower numbers of SM B cells had significantly fewer numbers of IgA+ SM B cells, as compared to those subjects with the infection only phenotype (mean 2.1% ± 2.8 for 46 Group 2 subjects vs. 3.6% ± 3.9% of B cells for 43 Group 1 subjects; P = 0.03) (data not shown).

### IFN-γ, CXCL9, and BAFF

**Figure 4.**
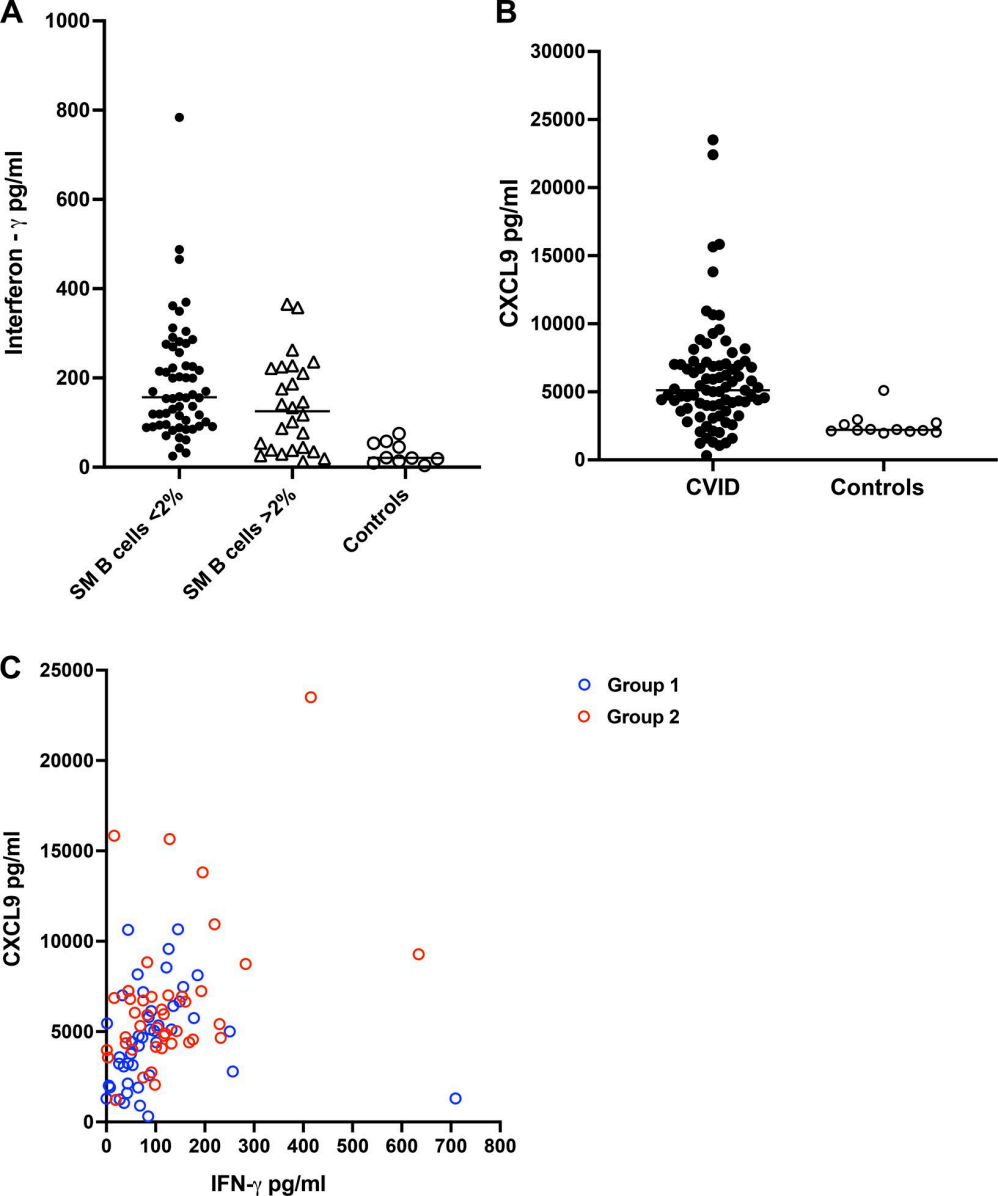
**Serum IFN-γ is increased in CVID subjects with very low isotype switched memory B cells; these subjects also have increased CXCL9, and more inflammatory complications. (A)** Serum IFN-γ and isotype SM B cells. Serum IFN-γ was significantly increased in sera of 88 CVID subjects with the fewest numbers of circulating isotype SM B cells, <2% (Mann–Whitney test, P = 0.03). 10 serums from normal controls are included. **(B)** Increased CXCL9. CVID subjects (*n* = 94) had increased serum CXCL9 as compared to 12 healthy controls (Mann–Whitney test P < 0.001). **(C)** Serum IFN-γ and CXCL9. For 89 CVID subjects, CXCL9 and IFN-γ levels were significantly correlated (Spearman test 4 = 0.4556; P < 0.0001). Subjects in Group 1 (no inflammatory complications) are distinguished from those with these complications (Group 2).

We previously showed that circulating bacterial 16S rDNA in the serum of subjects with CVID was associated with markedly increased serum IFN-γ levels (17). In addition, when CVID mononuclear cells of patients with inflammatory complications were exposed to 16S ribosomal DNA (rDNA), they produced large amounts of this cytokine in vitro (17, 18). Increased serum IFN-γ was also the case for the subjects studied here, particularly for those subjects with fewer isotype SM B cells. While CVID subjects of both groups had more serum IFN-γ than controls (P = 0.0001), subjects with <2% of SM B cells had more serum IFN-γ than those with more than this number (P = 0.05) (using the previously suggested cutoff of 2%) (9) (Fig. 4 A). We also noted that as a measure of overall increased serum IFN-γ levels in CVID, the serum chemokine CXCL9 was also significantly increased in CVID subjects as compared to control sera (P < 0.0001) (Fig. 4 B). As one would expect, the level of CXCL9 was closely related to the level of IFN-γ in the same CVID samples (P = 0.0001). This is shown for the patients, again divided into those with (Group 2) or without (Group 1) inflammatory complications (Fig. 4 C). (Subjects with inflammatory complications had increased CXCL9 as compared to those with the infection only phenotype, P = 0.01.)

**Figure 5.**
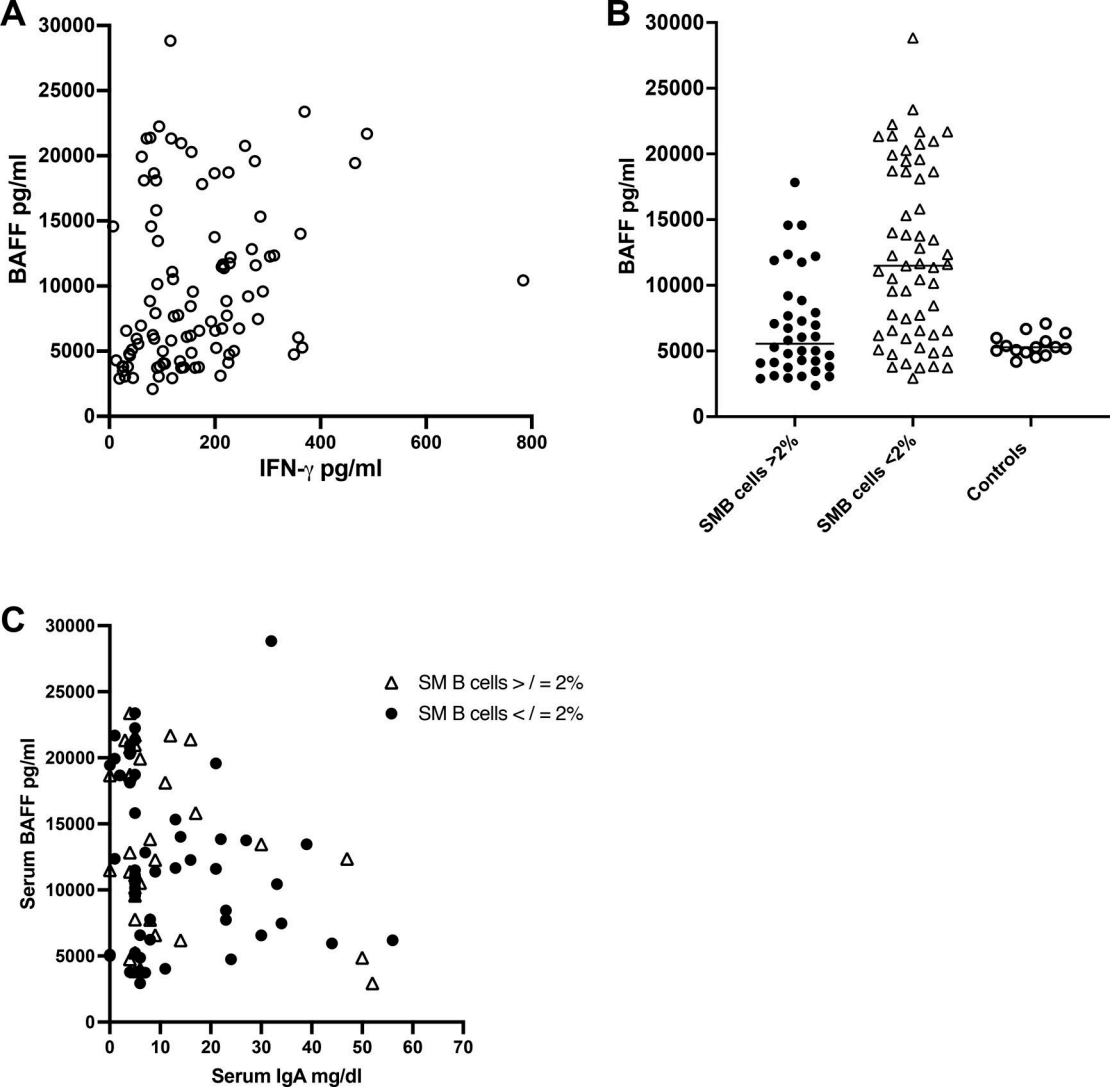
**The Serum BAFF level is related to IFN-γ content, is increased in those with fewest SMB cells, and the lowest serum IgA levels. (A)** BAFF and IFN-γ. BAFF was significantly related to the amount of IFN-γ in sera of 98 paired CVID sera (Spearman r = −0.3120, P = 0.002). **(B)** BAFF and isotype SM B cells. Serum BAFF was significantly increased with CVID subjects with very reduced SM B cells, <2% as compared to those subjects with 2% or more of these B cells (Mann–Whitney test, P = 0.0001) or normal controls. **(C)** BAFF and serum IgA. BAFF levels were inversely related to baseline serum IgA in 95 paired CVID serum samples (Spearman correlation, r = −0.2065; P = 0.04).

In other publications, IFN-γ has been shown to stimulate monocyte, myeloid, and T cell production of BAFF (27, 28). As we had shown previously that the serum of subjects with CVID commonly contains increased amounts of BAFF (24, 25), we also examined this cytokine in the sera of this CVID cohort. First, we show here that overall, serum BAFF concentrations were also significantly correlated with serum IFN-γ concentrations in the same CVID samples (P = 0.002) (Fig. 5 A). Second, for subjects with SM B cells of <2%, the median serum BAFF was 11,486 pg/ml, while for those with SM B cells >2%, the median BAFF was 5,549 pg/ml (P = <0.0001) (Fig. 5 B), possibly not surprising as these sera also contained increased IFN-γ concentrations. In fact, BAFF levels overall in CVID sera were closely related to the numbers of isotype SM B cells; P = 0.0013 (data not shown). Again suggesting the potential positive role of IgA, there was also an inverse correlation between serum BAFF concentrations and baseline serum IgA levels (P = 0.02) (Fig. 5 C).

## Discussion

One of the central questions regarding CVID has been why a significant proportion of patients have at diagnosis, or later develop, severe inflammatory and/or autoimmune complications, in spite of receiving the same treatment with substantial doses of immune globulin and antibiotics as needed. While genetics has supplied some of the answers, about 70% of subjects still do not have a known genetic basis (12, 13, 29). For this reason, we have been pursuing additional means to unravel the inflammatory pathways that underlie what is often termed the “noninfectious” complications of CVID. In previous work, seeking an unbiased approach, we demonstrated that a hallmark of the subjects with inflammatory/autoimmune complications was an IFN-γ mRNA signature, with a number of genes in this pathway being upregulated; in addition, we noted expanded numbers of IFN-γ secreting ILCs in blood and mucosal tissues of these subjects (14, 15, 30). To seek to explain these observations, and the cause of the IFN-γ elevation, we later identified that these subjects also had increased mucosal bacterial transcytosis, with high serum levels of 16S bacterial ribosomal DNA (rDNA), from species identified of gastrointestinal origin (17). This phenomenon was also associated with elevation of corresponding markers of systemic inflammation (lipopolysaccharide-binding protein, sCD14) as well as specific markers of gut mucosal barrier defects (zonulin and intestinal fatty acid binding protein (I-FABP)). We also showed that these biomarkers were a particular characteristic of subjects with very low numbers of circulating isotype SM B cells (17), previously also found to be characteristic of CVID subjects with greater risk of inflammatory/autoimmune complications (9, 10, 18). Bacterial transcytosis has previously been suggested to occur in CVID, as endotoxin in plasma and increased lipopolysaccharide have been identified, along with functional impairments of CD4 T cell proliferation, with increased programmed death 1 expression, as well as serum and plasma markers of systemic immune activation, sCD14 and sCD25 (31, 32, 33). Several studies have also noted dysbiosis with reduced stool microbial diversity and increases of selected microbial species in CVID, associated with elevated markers of immune activation in subjects with these inflammatory conditions. The acquisition of selected bacterial strains, potentially in the absence of IgA, could also promote the observed inflammatory phenotype (33, 34, 35).

As the loss of isotype-switched B cells in peripheral blood suggested to us that the greater losses of serum or mucosal IgA may be connected to the mucosal bacterial transcytosis, resulting in immune activation and the IFN-γ RNA signature, we then examined this connection. Although deficient serum IgA is a general characteristic of the CVID immune defect, many subjects still have at least some detectable levels in peripheral blood. While we are not able to quantitate levels of mucosal IgA, we show here that the amounts of residual baseline serum IgA is significantly associated with the level of microbial 16S ribosomal DNA (rDNA) in blood and that the baseline level of serum IgA, and even the number of circulating IgA+ SM B cells, can serve as biomarkers for bacterial transcytosis. This also agrees with previous work, which showed that the lowest levels of IgA were noted in the duodenal tissues of subjects with enteropathy (35) and in another study, lower levels of plasma IgA in CVID subjects were also associated with reduced stool microbial alpha diversity (33) potentially contributing to the inflammatory phenotype. These observations are also compatible with our previous work, in which retained serum IgA and increased numbers of IgA+ SM B cells in CVID subjects with autoimmune cytopenias were associated with lower serum endotoxin levels (36). In fact, in the CVID subjects with autoimmune cytopenias in this study, CD27^+^IgA^+^ SM B cells were virtually absent in peripheral blood (36). In addition, CVID patients with very rare circulating IgA^+^ SM B cells were also characterized by an increase in VH4-34^+^IgG^+^ B cells that were reported to recognize commensal bacteria, further illustrating the impaired gut microbiota containment in these patients (36, 37). As a consequence, increased commensal bacteria transcytosis in CVID patients with very low or absent serum IgA may trigger inflammatory immune responses, potentially also related to the hyperplastic but inefficient germinal center responses in these patients, associated with enhanced T follicular helper numbers and decreased regulatory T (Treg) cell numbers and function (36). Since Treg cells play an important role in preventing the accumulation of autoreactive naïve B cells in the periphery (38, 39, 40), it may not be surprising that elevated frequencies of autoreactive clones were previously detected in the mature naïve B cell compartment of CVID patients in which Treg cell functions were compromised (25). Together, these observations may also help to explain why CVID subjects with the lowest numbers of isotype SM B cells, with greater losses of IgA, may be more likely to have inflammatory/autoimmune complications. While some subjects with CVID have some retained IgM, and if secreted, this Ig could play a protective role in gastrointestinal mucosal tissues, we found no relationship between serum IgM and bacterial DNA levels in blood. In addition, one study suggested that the IgM produced in CVID may possess an autoimmune potential, with the ability to bind to cellular antigens (41).

Our previous work also showed that CVID subjects with increased microbial ribosomal DNA (rDNA) in blood also had increased serum IFN-γ and that adding microbial ribosomal DNA (rDNA) in vitro to mononuclear cells of subjects with known inflammatory conditions led to the production of more IFN-γ in these cultures (17). Here we show that, as one might expect, subjects with the lowest numbers of isotype SM B cells, those known to be more likely to have these complications (9, 10), also have significantly more IFN-γ in their serum. IFN-γ was previously shown in other studies to stimulate the production of BAFF by monocytes and myeloid cells (27, 28), an observation of potential importance in lymphoid hypertrophy and autoimmunity (42). Perhaps, in line with this, serum BAFF levels in CVID were also directly correlated with serum IFN-γ levels in paired serum samples. We previously showed that the serum of subjects with CVID contains large amounts of BAFF (24, 25), a factor also associated with pulmonary B cell infiltrates with germinal center formation in the lungs of our CVID patients (43). Here we show that serum BAFF concentrations were also inversely correlated with serum IgA concentrations, suggesting that the mucosal defects may be connected to the overproduction of these inflammatory signals. Decreased numbers of B cells, and especially memory B cells that express several receptors for BAFF (i.e., BAFF receptor and TACI), which consume this growth factor, could also result in an increase in serum BAFF concentrations (44). In agreement with this scenario, we found that CVID subjects with the lowest numbers of isotype SM B cells are also those subjects with significantly more serum BAFF.

In summary, the data from this large cohort of CVID subjects provide an overall framework to better understand some of the poorly understood aspects of this complex immune defect. Why do those subjects with the lowest numbers of isotype SM B cells have the greatest risk of inflammatory and autoimmune complications? And what are the mediators of this inflammation? Here we show that bacterial transcytosis due to mucosal barrier defects and loss of B cell isotype class-switched B cells, and therefore IgA production, promotes an IFN-γ signature, leading to inflammation, which in turn activates also numerous mediators of inflammation, one of which is excess BAFF, which may promote survival and proliferation of immature B cells. While IgA from normal donors might not address the dysbiotic microbial species found in subjects with CVID (34), polyclonal IgA supplementation, in parallel with IgG replacement, (for subjects who have not developed anti-IgA antibodies) could represent a novel therapeutic strategy to restore proper gut mucosal barriers and commensal bacteria containment, which may then thwart inflammation and autoimmunity in these patients.

## Materials and methods

### Patients

Patients with CVID (59 females and 55 males, median age of 55, range 24–90) were enrolled with informed consent in a Mount Sinai Institutional Review Board–approved protocol designed to investigate human B cell defects. Patients were diagnosed by standard criteria, including reduced serum IgG, IgA, and/or IgM with loss of antibody to vaccine protein and polysaccharide antigens (1). Baseline patient characteristics showed a mean of IgG = 200 mg/dl (range 9–593 mg/dl), IgA = 16.5 mg/dl (0–179 mg/dl range), and IgM = 46.1 mg/dl (range 0–81 mg/dl). The age range was 22–72 years of age. The inflammatory medical complications experienced by each subject were categorized as previously outlined: infections only or subjects with autoimmunity, interstitial lung disease, granulomatous disease, lymphoid hyperplasia/splenomegaly, gastrointestinal enteropathy, liver disease, and/or lymphoma (4, 5). Control subjects were healthy adults, 13 females and 8 males, ages 22–64.

### B cell populations

B cell populations from patients were examined as previously described (9, 10), identifying total B cells, CD27^+^ memory B cells, and isotype SM cells (CD27+, IgD−IgM−). Separately, we also identified IgA+CD27+ IgD−IgM− B cells from patients and controls. Subjects were stratified into two groups, as those with 2% or more SM B cells and those with fewer than this number, as previously published (9).

### Genetic evaluations

Genetic evaluation was done by whole-exome sequencing as previously described (11, 12, 13, 45, 46). In some cases, patient exomes were also examined for mutations in a panel of 429 genes associated with primary immune deficiency disease (Invitae Diagnostics).

### Quantitative PCR for bacterial 16S ribosomal DNA (rDNA)

Serum from patients were drawn into 6-ml gold top rubber-sealed sterile Vacutainer SST II tubes (BD Diagnostics) as described (17). The tubes maintained in the upright position until the serum was aseptically removed in a sterile hood. Bacterial DNA analysis was performed as previously described, using a DNA standard prepared from *Escherichia coli* competent cells (Qiagen); concentrations were measured by NanoDrop. Using the *E. coli* genome length (4,700,000), the number of *E. coli* DNA copies was calculated as described (17), and serial dilutions of this standard were used to define DNA copy numbers in a standard curve.

### Serum IFN-γ and BAFF

Serum IFN-γ was determined using a human IFN-γ ELISA (R&D Systems) using dilutions of (1:2,500, 1:1,000, and 1:10,000) (R&D Systems). Serum CXCL9 was also examined in the same sera by ELISA (R&D Systems); both are expressed in pg/ml. Serum BAFF was quantitated in serum, diluted 1:2 by ELISA using a commercial kit (Axxora LLC), and expressed in pg/ml.

### Statistical analysis

The nonparametric Mann–Whitney test was used to compare bacterial DNA and class SM B cells between subjects with inflammatory complications from those without these medical problems. This test was also used to compare serum BAFF, IFN-γ, and CXCL9 levels in patient groups and controls. For determining the correlations between the paired variables, bacterial DNA, IFN-γ, CXCL9, BAFF, serum IgA, and isotype SM B cells, the nonparametric Spearman rank correlation test was used. In all tests, P values <0.05 were considered significant. All calculations were performed using Prism GraphPad software.

### Study approval

This study was approved by the institutional review board of the Icahn School of Medicine at Mount Sinai and was carried out in accordance with the Code of Ethics of the World Medical Association (Declaration of Helsinki). Written informed consent was received from participants prior to inclusion in the study.

## Data Availability

The data underlying these results and methods of analysis are available on request.

## References

[1] Bonilla, F.A., I. Barlan, H. Chapel, B.T. Costa-Carvalho, C. Cunningham-Rundles, M.T. de la Morena, F.J. Espinosa-Rosales, L. Hammarström, S. Nonoyama, I. Quinti, . 2016. International consensus document (ICON): Common variable immunodeficiency disorders. J. Allergy Clin. Immunol. Pract. 4:38–59. 10.1016/j.jaip.2015.07.025 26563668 PMC4869529

[2] Seidel, M.G., G. Kindle, B. Gathmann, I. Quinti, M. Buckland, J. van Montfrans, R. Scheible, S. Rusch, L.M. Gasteiger, B. Grimbacher, . 2019. The European society for immunodeficiencies (ESID) registry working definitions for the clinical diagnosis of inborn errors of immunity. J. Allergy Clin. Immunol. Pract. 7:1763–1770. 10.1016/j.jaip.2019.02.004 30776527

[3] Tangye, S.G., W. Al-Herz, A. Bousfiha, C. Cunningham-Rundles, J.L. Franco, S.M. Holland, C. Klein, T. Morio, E. Oksenhendler, C. Picard, . 2022. Human inborn errors of immunity: 2022 Update on the classification from the international union of immunological societies expert committee. J. Clin. Immunol. 42:1473–1507. 10.1007/s10875-022-01289-3 35748970 PMC9244088

[4] Chapel, H., M. Lucas, M. Lee, J. Bjorkander, D. Webster, B. Grimbacher, C. Fieschi, V. Thon, M.R. Abedi, and L. Hammarstrom. 2008. Common variable immunodeficiency disorders: Division into distinct clinical phenotypes. Blood. 112:277–286. 10.1182/blood-2007-11-124545 18319398

[5] Resnick, E.S., E.L. Moshier, J.H. Godbold, and C. Cunningham-Rundles. 2012. Morbidity and mortality in common variable immune deficiency over 4 decades. Blood. 119:1650–1657. 10.1182/blood-2011-09-377945 22180439 PMC3286343

[6] Cunningham-Rundles, C., and C. Bodian. 1999. Common variable immunodeficiency: Clinical and immunological features of 248 patients. Clin. Immunol. 92:34–48. 10.1006/clim.1999.4725 10413651

[7] Ho, H.E., and C. Cunningham-Rundles. 2020. Non-infectious complications of common variable immunodeficiency: Updated clinical spectrum, sequelae, and insights to pathogenesis. Front Immunol. 11:149. 10.3389/fimmu.2020.00149 32117289 PMC7025475

[8] Bez, P., B. Smits, C. Geier, A. Hirsch, A. Caballero de Oyteza, M. Proietti, B. Grimbacher, M. Wolkewitz, S. Goldacker, and K. Warnatz. 2025. Uncovering risk factors of premature mortality in common variable immunodeficiency (CVID). J. Allergy Clin. Immunol. Pract. 13:1201–1209.e10. 10.1016/j.jaip.2025.03.009 40090481

[9] Wehr, C., T. Kivioja, C. Schmitt, B. Ferry, T. Witte, E. Eren, M. Vlkova, M. Hernandez, D. Detkova, P.R. Bos, . 2008. The EUROclass trial: Defining subgroups in common variable immunodeficiency. Blood. 111:77–85. 10.1182/blood-2007-06-091744 17898316

[10] Sanchez-Ramon, S., L. Radigan, J.E. Yu, S. Bard, and C. Cunningham-Rundles. 2008. Memory B cells in common variable immunodeficiency: Clinical associations and sex differences. Clin. Immunol. 128:314–321. 10.1016/j.clim.2008.02.013 18620909 PMC2692232

[11] Maffucci, P., C.A. Filion, B. Boisson, Y. Itan, L. Shang, J.L. Casanova, and C. Cunningham-Rundles. 2016. Genetic diagnosis using whole exome sequencing in common variable immunodeficiency. Front Immunol. 7:220. 10.3389/fimmu.2016.00220 27379089 PMC4903998

[12] Abolhassani, H., L. Hammarstrom, and C. Cunningham-Rundles. 2020. Current genetic landscape in common variable immune deficiency. Blood. 135:656–667. 10.1182/blood.2019000929 31942606 PMC7046605

[13] Cunningham-Rundles, C., J.L. Casanova, and B. Boisson. 2023. Genetics and clinical phenotypes in common variable immunodeficiency. Front Genet. 14:1272912. 10.3389/fgene.2023.1272912 38274105 PMC10808799

[14] Park, J., I. Munagala, H. Xu, D. Blankenship, P. Maffucci, D. Chaussabel, J. Banchereau, V. Pascual, and C. Cunningham-Rundles. 2013. Interferon signature in the blood in inflammatory common variable immune deficiency. PLoS One. 8:e74893. 10.1371/journal.pone.0074893 24069364 PMC3775732

[15] Cols, M., A. Rahman, P.J. Maglione, Y. Garcia-Carmona, N. Simchoni, H.M. Ko, L. Radigan, A. Cerutti, D. Blankenship, V. Pascual, and C. Cunningham-Rundles. 2016. Expansion of inflammatory innate lymphoid cells in patients with common variable immune deficiency. J. Allergy Clin. Immunol. 137:1206–1215 e6. 10.1016/j.jaci.2015.09.013 26542033 PMC4866594

[16] Panda, S.K., and M. Colonna. 2019. Innate lymphoid cells in mucosal immunity. Front Immunol. 10:861. 10.3389/fimmu.2019.00861 31134050 PMC6515929

[17] Ho, H.E., L. Radigan, G. Bongers, A. El-Shamy, and C. Cunningham-Rundles. 2021. Circulating bioactive bacterial DNA is associated with immune activation and complications in common variable immunodeficiency. JCI Insight. 6:e144777. 10.1172/jci.insight.144777 34622805 PMC8525635

[18] Ho, H.E., L. Radigan, J. Qi, V. Roudko, M.J. Storek, J.H. Lee, R. Fuleihan, K. Sullivan, S. Kim-Schulze, and C. Cunningham-Rundles. 2025. Bruton tyrosine kinase modulates systemic immune activation to bacterial translocation in primary antibody deficiencies. J. Allergy Clin. Immunol. 156:1693–1705.e5. 10.1016/j.jaci.2025.09.019 41033468

[19] Wilmore, J.R., B.T. Gaudette, D. Gomez Atria, T. Hashemi, D.D. Jones, C.A. Gardner, S.D. Cole, A.M. Misic, D.P. Beiting, and D. Allman. 2018. Commensal microbes induce serum IgA responses that protect against polymicrobial sepsis. Cell Host Microbe. 23:302–311.e3. 10.1016/j.chom.2018.01.005 29478774 PMC6350773

[20] Bunker, J.J., T.M. Flynn, J.C. Koval, D.G. Shaw, M. Meisel, B.D. McDonald, I.E. Ishizuka, A.L. Dent, P.C. Wilson, B. Jabri, . 2015. Innate and adaptive humoral responses coat distinct commensal bacteria with immunoglobulin A. Immunity. 43:541–553. 10.1016/j.immuni.2015.08.007 26320660 PMC4575282

[21] Bunker, J.J., S.A. Erickson, T.M. Flynn, C. Henry, J.C. Koval, M. Meisel, B. Jabri, D.A. Antonopoulos, P.C. Wilson, and A. Bendelac. 2017. Natural polyreactive IgA antibodies coat the intestinal microbiota. Science 358:eaan6619. 10.1126/science.aan6619 28971969 PMC5790183

[22] Bioley, G., J. Monnerat, M. Lotscher, C. Vonarburg, A. Zuercher, and B. Corthesy. 2017. Plasma-derived polyreactive secretory-like IgA and IgM opsonizing Salmonella enterica typhimurium reduces invasion and gut tissue inflammation through agglutination. Front Immunol. 8:1043. 10.3389/fimmu.2017.01043 28900429 PMC5581814

[23] Conrey, P.E., L. Denu, K.C. O'Boyle, I. Rozich, J. Green, J. Maslanka, J.-B. Lubin, T. Duranova, B.L. Haltzman, L. Gianchetti, . 2023. IgA deficiency destabilizes homeostasis toward intestinal microbes and increases systemic immune dysregulation. Sci. Immunol. 8:eade2335. 10.1126/sciimmunol.ade2335 37235682 PMC11623094

[24] Knight, A.K., L. Radigan, T. Marron, A. Langs, L. Zhang, and C. Cunningham-Rundles. 2007. High serum levels of BAFF, APRIL, and TACI in common variable immunodeficiency. Clin. Immunol. 124:182–189. 10.1016/j.clim.2007.04.012 17556024 PMC2491330

[25] Romberg, N., N. Chamberlain, D. Saadoun, M. Gentile, T. Kinnunen, Y.S. Ng, M. Virdee, L. Menard, T. Cantaert, H. Morbach, . 2013. CVID-associated TACI mutations affect autoreactive B cell selection and activation. J. Clin. Invest. 123:4283–4293. 10.1172/JCI69854 24051380 PMC3786721

[26] Maglione, P.J., J.R. Overbey, and C. Cunningham-Rundles. 2015. Progression of common variable immunodeficiency interstitial lung disease accompanies distinct pulmonary and laboratory findings. J. Allergy Clin. Immunol. Pract. 3:941–950. 10.1016/j.jaip.2015.07.004 26372540 PMC4641811

[27] Woo, S.J., J. Im, J.H. Jeon, S.S. Kang, M.H. Lee, C.H. Yun, E.-Y. Moon, M.K. Song, H.-H. Kim, and S.H. Han. 2013. Induction of BAFF expression by IFN-gamma via JAK/STAT signaling pathways in human intestinal epithelial cells. J. Leukoc. Biol. 93:363–368. 10.1189/jlb.0412210 23271704

[28] Scapini, P., Y. Hu, C.L. Chu, T.S. Migone, A.L. Defranco, M.A. Cassatella, and C.A. Lowell. 2010. Myeloid cells, BAFF, and IFN-gamma establish an inflammatory loop that exacerbates autoimmunity in Lyn-deficient mice. J. Exp. Med. 207:1757–1773. 10.1084/jem.20100086 20624892 PMC2916124

[29] Rojas-Restrepo, J., A. Caballero-Oteyza, K. Huebscher, H. Haberstroh, M. Fliegauf, B. Keller, R. Kobbe, K. Warnatz, S. Ehl, M. Proietti, and B. Grimbacher. 2021. Establishing the molecular diagnoses in a cohort of 291 patients with predominantly antibody deficiency by targeted next-generation sequencing: Experience from a monocentric study. Front Immunol. 12:786516. 10.3389/fimmu.2021.786516 34975878 PMC8718408

[30] Cols, M., A. Rahman, P.J. Maglione, Y. Garcia-Carmona, N. Simchoni, H.-B.M. Ko, L. Radigan, A. Cerutti, D. Blankenship, V. Pascual, and C. Cunningham-Rundles. 2016. Expansion of inflammatory innate lymphoid cells in patients with common variable immune deficiency. J. Allergy Clin. Immunol. 137:1206–1215.e6. 10.1016/j.jaci.2015.09.013 26542033 PMC4866594

[31] Perreau, M., S. Vigano, F. Bellanger, C. Pellaton, G. Buss, D. Comte, T. Roger, C. Lacabaratz, P.-A. Bart, Y. Levy, and G. Pantaleo. 2014. Exhaustion of bacteria-specific CD4 T cells and microbial translocation in common variable immunodeficiency disorders. J. Exp. Med. 211:2033–2045. 10.1084/jem.20140039 25225461 PMC4172212

[32] Le Coz, C., B. Bengsch, C. Khanna, M. Trofa, T. Ohtani, B.E. Nolan, S.E. Henrickson, M.P. Lambert, T.O. Kim, J.M. Despotovic, . 2019. Common variable immunodeficiency-associated endotoxemia promotes early commitment to the T follicular lineage. J. Allergy Clin. Immunol. 144:1660–1673. 10.1016/j.jaci.2019.08.007 31445098 PMC6900457

[33] Jorgensen, S.F., M. Troseid, M. Kummen, J.A. Anmarkrud, A.E. Michelsen, L.T. Osnes, K. Holm, M.L. Høivik, A. Rashidi, C.P. Dahl, . 2016. Altered gut microbiota profile in common variable immunodeficiency associates with levels of lipopolysaccharide and markers of systemic immune activation. Mucosal Immunol. 9:1455–1465. 10.1038/mi.2016.18 26982597

[34] Hajjar, J., A.Rehman, A.Hamdi, and I.Fuss. 2025. Navigating the complexities of common variable immunodeficiency enteropathy: From established therapies to emerging interventions. Immunol. Allergy Clin. North Am.45:267–285. 10.1016/j.iac.2025.01.00540287172

[35] Shulzhenko, N., X. Dong, D. Vyshenska, R.L. Greer, M. Gurung, S. Vasquez-Perez, E. Peremyslova, S. Sosnovtsev, M. Quezado, M. Yao, . 2018. CVID enteropathy is characterized by exceeding low mucosal IgA levels and interferon-driven inflammation possibly related to the presence of a pathobiont. Clin. Immunol. 197:139–153. 10.1016/j.clim.2018.09.008 30240602 PMC6289276

[36] Romberg, N., C. Le Coz, S. Glauzy, J.N. Schickel, M. Trofa, B.E. Nolan, M. Paessler, M.L. Xu, M.P. Lambert, S.A. Lakhani, . 2019. Patients with common variable immunodeficiency with autoimmune cytopenias exhibit hyperplastic yet inefficient germinal center responses. J. Allergy Clin. Immunol. 143:258–265. 10.1016/j.jaci.2018.06.012 29935219 PMC6400323

[37] Schickel, J.N., S. Glauzy, Y.S. Ng, N. Chamberlain, C. Massad, I. Isnardi, N. Katz, G. Uzel, S.M. Holland, C. Picard, . 2017. Self-reactive VH4-34-expressing IgG B cells recognize commensal bacteria. J. Exp. Med. 214:1991–2003. 10.1084/jem.20160201 28500047 PMC5502416

[38] Kinnunen, T., N. Chamberlain, H. Morbach, J. Choi, S. Kim, J. Craft, L. Mayer, C. Cancrini, L. Passerini, R. Bacchetta, . 2013. Accumulation of peripheral autoreactive B cells in the absence of functional human regulatory T cells. Blood. 121:1595–1603. 10.1182/blood-2012-09-457465 23223361 PMC3587322

[39] Chen, J.W., J.N. Schickel, N. Tsakiris, J. Sng, F. Arbogast, D. Bouis, D. Parisi, R. Gera, J.M. Boeckers, F.R. Delmotte, . 2022. Positive and negative selection shape the human naive B cell repertoire. J. Clin. Invest. 132:e150985. 10.1172/JCI150985 34813502 PMC8759783

[40] Sng, J., B. Ayoglu, J.W. Chen, J.N. Schickel, E.M.N. Ferre, S. Glauzy, N. Romberg, M. Hoenig, C. Cunningham-Rundles, P.J. Utz, . 2019. AIRE expression controls the peripheral selection of autoreactive B cells. Sci. Immunol. 4:eaav6778. 10.1126/sciimmunol.aav6778 30979797 PMC7257641

[41] Le Coz, C., M. Trofa, D.L. Butler, S. Yoon, T. Tian, W. Reid, E. Cruz Cabrera, A.V.C. Knox, C. Khanna, K.E. Sullivan, . 2024. The common variable immunodeficiency IgM repertoire narrowly recognizes erythrocyte and platelet glycans. J. Allergy Clin. Immunol. 154:778–791.e9. 10.1016/j.jaci.2024.04.018 38692308 PMC11380600

[42] Mackay, F., P.A. Silveira, and R. Brink. 2007. B cells and the BAFF/APRIL axis: Fast-forward on autoimmunity and signaling. Curr. Opin. Immunol. 19:327–336. 10.1016/j.coi.2007.04.008 17433868

[43] Maglione, P.J., G. Gyimesi, M. Cols, L. Radigan, H.M. Ko, T. Weinberger, B.H. Lee, E.K. Grasset, A.H. Rahman, A. Cerutti, and C. Cunningham-Rundles. 2019. BAFF-driven B cell hyperplasia underlies lung disease in common variable immunodeficiency. JCI Insight. 4:e122728. 10.1172/jci.insight.122728 30843876 PMC6483510

[44] Cantaert, T., J.N. Schickel, J.M. Bannock, Y.S. Ng, C. Massad, F.R. Delmotte, N. Yamakawa, S. Glauzy, N. Chamberlain, T. Kinnunen, . 2016. Decreased somatic hypermutation induces an impaired peripheral B cell tolerance checkpoint. J. Clin. Invest. 126:4289–4302. 10.1172/JCI84645 27701145 PMC5096912

[45] Picard, C., H. Bobby Gaspar, W. Al-Herz, A. Bousfiha, J.L. Casanova, T. Chatila, Y.J. Crow, C. Cunningham-Rundles, A. Etzioni, J.L. Franco, . 2018. International union of immunological societies: 2017 primary immunodeficiency diseases committee report on inborn errors of immunity. J. Clin. Immunol. 38:96–128. 10.1007/s10875-017-0464-9 29226302 PMC5742601

[46] Maffucci, P., B. Bigio, F. Rapaport, A. Cobat, A. Borghesi, M. Lopez, E. Patin, A. Bolze, L. Shang, M. Bendavid, . 2019. Blacklisting variants common in private cohorts but not in public databases optimizes human exome analysis. Proc. Natl. Acad. Sci. USA. 116:950–959. 10.1073/pnas.1808403116 30591557 PMC6338851

